# Sustained high expression of multiple APOBEC3 cytidine deaminases in systemic lupus erythematosus

**DOI:** 10.1038/s41598-021-87024-1

**Published:** 2021-04-12

**Authors:** Danielle Perez-Bercoff, Hélène Laude, Morgane Lemaire, Oliver Hunewald, Valérie Thiers, Marco Vignuzzi, Hervé Blanc, Aurélie Poli, Zahir Amoura, Vincent Caval, Rodolphe Suspène, François Hafezi, Alexis Mathian, Jean-Pierre Vartanian, Simon Wain-Hobson

**Affiliations:** 1 Department of Infection and Immunity, Luxembourg Institute of Health, 29 rue Henri Koch, 4354 Esch-sur-Alzette, Luxembourg; 2 ICAReB Platform, 28 rue du Docteur Roux, 75724 Paris Cedex 15, France; 3 Molecular Retrovirology Unit, UMR 3569, Institut Pasteur, CNRS, 28 rue du Dr. Roux, 75724 Paris cedex 15, France; 4 Viral Populations and Pathogenesis Unit, UMR 3569, CNRS, Institut Pasteur, 28 rue du Dr. Roux, 75724 Paris Cedex 15, France; 5 Sorbonne Université, Assistance Publique–Hôpitaux de Paris, Groupement Hospitalier Pitié–Salpêtrière, French National Referral Center for Systemic Lupus Erythematosus, Antiphospholipid Antibody Syndrome and Other Autoimmune Disorders, Service de Médecine Interne 2, Institut E3M, Inserm UMRS, Centre D’Immunologie Et Des Maladies Infectieuses (CIMI-Paris), Paris, France; 6 Departement de Virologie, Institut Pasteur, 28 rue du Dr. Roux, 75724 Paris Cedex 15, France

**Keywords:** Cancer, Genetics, Immunology

## Abstract

APOBEC3 (A3) enzymes are best known for their role as antiviral restriction factors and as mutagens in cancer. Although four of them, A3A, A3B, A3F and A3G, are induced by type-1-interferon (IFN-I), their role in inflammatory conditions is unknown. We thus investigated the expression of *A3*, and particularly *A3A* and *A3B* because of their ability to edit cellular DNA, in Systemic Lupus Erythematosus (SLE), a chronic inflammatory disease characterized by high IFN-α serum levels. In a cohort of 57 SLE patients, *A3A* and *A3B,* but also *A3C* and *A3G,* were upregulated ~ 10 to 15-fold (> 1000-fold for *A3B*) compared to healthy controls, particularly in patients with flares and elevated serum IFN-α levels. Hydroxychloroquine, corticosteroids and immunosuppressive treatment did not reverse *A3* levels. The *A3AΔ3B* polymorphism, which potentiates *A3A*, was detected in 14.9% of patients and in 10% of controls, and was associated with higher *A3A* mRNA expression. *A3A* and *A3B* mRNA levels, but not *A3C* or *A3G*, were correlated positively with dsDNA breaks and negatively with lymphopenia. Exposure of SLE PBMCs to IFN-α in culture induced massive and sustained *A3A* levels by 4 h and led to massive cell death. Furthermore, the rs2853669 A > G polymorphism in the telomerase reverse transcriptase (TERT) promoter, which disrupts an Ets-TCF-binding site and influences certain cancers, was highly prevalent in SLE patients, possibly contributing to lymphopenia. Taken together, these findings suggest that high baseline *A3A* and *A3B* levels may contribute to cell frailty, lymphopenia and to the generation of neoantigens in SLE patients. Targeting *A3* expression could be a strategy to reverse cell death and the generation of neoantigens.

## Introduction

Apolipoprotein B mRNA-editing enzyme catalytic polypeptide-like 3 (APOBEC3 or A3) enzymes are a family of six cytidine deaminases (A3A-A3C, A3F-A3H) which deaminate cytidine (C) into uracil (U) on single stranded DNA (ssDNA)^1,2^. Uracil in genomic DNA can be read as a thymidine (T), resulting in C > T transitions. Alternatively, downstream processing of genomic uracil by the cellular Base Excision Repair (BER) machinery may correctly repair the mutation, lead to C > T transitions or C > G transversions depending on the polymerase involved, or generate double-stranded DNA breaks (DSBs)^3–5^. This latter mechanism has been shown to operate following deamination by AID, A3A and A3B^6–8^. A3A and A3B are mainly expressed in monocytes and B-cells respectively, while most other A3F and A3G predominate in T-cells^9^. A3 enzymes are a powerful intrinsic immune mechanism shielding cells against a range of viruses and retro-elements^10–14^. In keeping with their antiviral function, four of the seven A3 enzymes (A3A, A3B, A3F and A3G) are strongly induced by IFN-α^9,15,16^.

For unknown reasons A3A and A3B are able to edit chromosomal DNA^8,17,18^. Accordingly, clusters of C > T edits (or G > A on the opposite strand) attributed to A3A and A3B are the most common somatic mutations found in numerous cancer genomes^6,7,19,20^. As such, A3A and A3B are now acknowledged as DNA mutators. Recent studies suggest that A3A is the main enzyme responsible for APOBEC-induced mutations in some cancers for its higher deamination activity and propensity to generate DSBs^21–23^. Epidemiological studies have linked polymorphisms within *A3A* and/or *A3B* to different cancers^24–27^. Single Nucleotide Polymorphisms (SNPs) in A3A have been associated with liver, pancreas, bladder and lung cancer not related to smoking^24–27^. A 29 kbase deletion between exon 5 of *A3A* and exon 8 of *A3B* (*A3AΔ3B*) is a prevalent polymorphism in South East Asia and is almost fixed in Oceania^28^. This deletion generates a chimeric A3A–A3B transcript where the *A3A* coding sequence is unaffected but terminates with the *A3B* 3′ untranslated region (3′UTR). This *A3AΔ3B* deletion is associated with increased A3A expression^28^. *A3AΔ3B* is overrepresented in breast, ovarian and liver cancers and was proposed as a cancer-susceptibility gene^6,28–33^. In homozygous breast cancer carriers, it was associated with increased immune activation leading to hypermutation^26^.

A3 enzymes, and particulary A3A, are also involved in the catabolism of mitochondrial (mt) DNA (mtDNA) leaked into the cytoplasm in response to stress, circumventing chronic immune stimulation and apoptosis^8,17,18,34,35^. Nevertheless, the role of APOBEC3 deaminases in chronic inflammatory conditions has not been investigated. Chronic inflammation is a fertile ground for malignant transformation. A recent study investigating the link between dystrophic epidermolysis bullosa, a rare skin disease characterized by fragile skin and continual inflammation, and the development of squamous cell carcinoma (SCC), has unambigously identified APOBEC-mutation signatures as the most likely mechanistic cause of inflammation-driven SCC^36^. We hypothesized that a similar link might exist between chronic inflammatory conditions characterized by high circulating Type-I Interferon (IFN-I) such as Systemic Lupus Erythematosus (SLE) and higher cancer incidence. SLE is a chronic, disabling disease characterized by high levels of IFN-α likely associated with gain-of function in cytosolic nucleic acid sensors and sustained oxidative stress accompanied by mtDNA lesions (increased 8-oxoG or 8-OHdG). Sustained oxidative stress and mtDNA lesions lead to DNA leakage into the cytosol and induce potent IFN-I responses. Other hallmarks of SLE are profound lymphopenia, improper clearance of apoptotic and necrotic cells and circulating auto-antibodies against DNA and nuclear components^37–41^. The pathogenesis of SLE is multifactorial, involving genetic, immunological and environmental factors^41^. There is currently no licensed drug targeting IFN-I or the IFN-I receptor in SLE^42^. SLE patients have increased incidence of hematological malignancies (non-Hodgkin’s lymphoma, leukemia) and certain solid cancers (vulva and cervix, thyroid, lung, liver), but, although controversial, a decreased risk of hormonal-sensitive cancers (breast, endometrial, prostate)^43,44^. Some oncogenic pathways such as Akt-1^45^ and Telomerase Reverse Transcriptase (TERT) are activated in SLE patients’ peripheral blood mononuclear cells (PBMCs)^46–49^. In cancer, these pathways are activated by mutations. Akt1 mutation E17K results in constitutive activation of the kinase and is considered a driver gene mutation^50^. Mutations in the TERT promoter generating novel binding sites for transcription factors of the E-twenty-six (Ets)/TCF family are a common mechanism of TERT reactivation^51–53^. Akt1 E17K and *TERT* promoter mutations conform to the A3A/A3B preferred target, i.e. TpCpW, where W stands for A or T^7,21^.

As A3A and A3B are induced by IFN-I, and because A3A contributes to catabolizing cytoplasmic mtDNA^8,18,34,35^, we hypothesized that these enzymes are upregulated in SLE in response to mtDNA leaked into the cytoplasm. Sustained A3A and A3B expression might however edit nuclear DNA, thereby contributing to lymphopenia during flares, but also generating neo-antigens which in turn fuel the auto-immune response against nuclear antigens and the mutational burden. In this study, we present experimental evidence that *A3A*, *A3B*, *A3C* and *A3G* mRNA expression levels are significantly upregulated in SLE patients compared to healthy controls, particularly in patients with severe disease. In the studied samples, A3 upregulation was independent of treatment. The *A3AΔ3B* polymorphism was detected more often in patients with severe SLE and was associated with higher *A3A* mRNA levels, but not with any of the other A3. *A3A* and *A3B* levels were also correlated with higher DSBs and with lower lymphocyte counts. When SLE-patient cells were exposed to IFN-α, recapitulating flares in vitro, they readily expressed massive amounts of A3A and died within 24 h. Furthermore, a common polymorphism (rs2853669 A > G) which decreases TERT expression^54–59^, was much more common in SLE patients than in healthy controls. These findings clearly point to a role for A3A and A3B in the pathogenesis of lupus and in inflammation-associated lymphopenia.

## Results and discussion

### Characteristics of the SLE patients

Patients’ baseline characteristics are described in Table 1. Mean (± standard deviation) age at sample collection was 32.9 ± 12.5 years. Mean disease duration was 8.8 ± 8. The majority of patients had a clinically and/or serologically active disease. Thirty patients suffered from severe flares, 11 from mild or moderate flares and 16 had no flares. The median (range) SELENA-SLE disease activity index (SLEDAI) score was 6 (0–32). Thirty (57%) patients had a positive Farr assay and 23 (40%) had low C3 serum level. Thirty-nine (68.4%) patients had serum auto-antibodies (14 patients had auto-antibodies against one ribonucleoprotein (RNP), SSA52/TRIM21, SSA/Ro60, SSB or Sm and 25 patients against two or more of the above nuclear auto-antigens). Eighteen (31.6%) SLE patients had no detectable auto-antibodies. Serum IFN-α levels were elevated in 33 (58%) patients, with a median of 2 IU/mL (0–201).

**Table 1:** Disease characteristics in SLE patients.

	Patients (N = 57)
Women	52 (91)
Age, years, mean ± SD	32.9 ± 12.5
Disease duration, years, mean ± SD	8.8 ± 8
SELENA–SLEDAI score, median (range)	6 (0–32)
SELENA–SLEDAI score = 0	11 (19)
SELENA–SLEDAI score > 0 to ≤ 5	11 (19)
SELENA–SLEDAI score > 6 to ≤ 10	20 (35)
SELENA–SLEDAI score > 10	15 (26)
No flare^a^	16 (28)
Mild/moderate flare^a^	11 (19)
Severe flare^a^	30 (52)
**Clinical involvement**	
Fever	16 (28)
Weight loss or anorexia	12 (21)
Lymphadenopathy	10 (17)
Active cutaneous lupus	24 (42)
Active lupus serositis	9 (16)
Active lupus arthritis	21 (37)
Active lupus nephropathy	11 (19)
Proliferative nephropathy (classe III or IV)	4 (7)
Membranous nephropathy (isolated classe V)	5 (9)
Active neuropsychiatric lupus	4 (7)
Lymphopenia	14 (24)
**Treatment regimen**	
Hydroxychloroquine use	44 (77)
Prednisone use	32 (56)
Prednisone ≥ 10 mg/j	18 (31)
Immunosuppressive agent use^b^	20 (35)
**Biological tests**	
Positive Farr test	30/53^c^ (57)
Positive anti-RNP Abs	24 (42)
Positive anti-Sm Abs	18 (31)
Positive anti-Ro/SSA 52 Abs	18 (31)
Positive anti-Ro/SSA 60 Abs	27 (47)
Positive anti-La/SSB Abs	6 (10)
Low C3	23/57^c^ (40)
Elevated serum IFNα level	33 (58)
Serum IFNα level, IU/mL, median (range)	2 (0–201)

Values are expressed as n (%), unless stated otherwise.

SD, standard deviation; SELENA–SLEDAI, Safety of Estrogens in Lupus Erythematosus: National Assessment version of the Systemic Lupus Erythematosus Disease Activity Index.

^a^Defined using SELENA flare index^62,63^.

^b^Excluding antimalarials and prednisone. Immunosuppressant therapy was methotrexate (MTX) for 12 (60%), mycophenolate mofetil (MMF) for 3 (15%) patients, azathioprine for 3 (15%), cyclophosphamide for 1 (5%) and adalimumab for 1 (5%).

^c^Positive assay/number of patients assessed.

Treatment for SLE is based on hydroxychloroquine (HCQ), glucocorticosteroids (Prednisone (PDN)), and immunomodulatory agents (methotrexate (MTX), mycophenolate mofetil (MMF), Cyclophosphamide (Cyc), Azathioprine (AZA))^60,61^. In our cohort, most SLE patients were treated (treated: 47 patients, no treatment: 10 patients) (Table 1).

### *A3A* and *A3B* are upregulated in SLE patients

**Figure 1:**
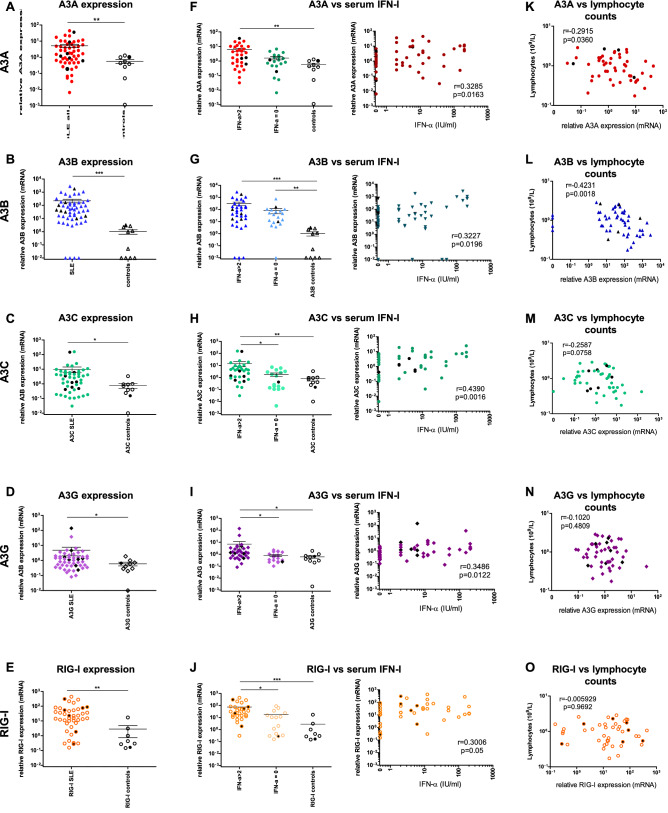
*A3* relative expression in a cohort of SLE patients. **A**–**D** Relative *A3A* (**A**), *A3B* (**B**), *A3C* (**C**) *A3G* (**D**) expression (mRNA) in SLE patients and in age- and sex-matched healthy controls). (**E**) Relative expression of RIG-I in SLE patients and in healthy controls. **F**–**J**. *A3A* (**F**) *A3B* (**G**), *A3C* (**H**), *A3G* (**I**) and *RIG-I* (**J**) expression in relation to serum IFN-I levels (IU/mL). **K**–**O**. Correlation of expression of *A3A* (**K**), *A3B* **(L**), *A3C* (**M**), *A3G* (**N**) and *RIG-I* (**O**) with lymphocyte counts**.** Target gene (*A3A*, *A3B*, *A3C*, *A3G* and *RIG-I*) levels were normalized to the geometric mean of housekeeping genes *RPL13A* and *GAPDH* according to the Pfaffl method^65^. Control C1 was used as calibrator for comparison and is therefore set to 1. The mean of duplicate measures ± SEM are reported. Patients with 2 wt *A3B* alleles are represented by colored symbols (red circles in panels **A** **F** and **K**, blue triangles in panels **B**, **G** and **L**, green circles in panels **C**, **H** and **M**, purple diamonds in panels **D**, **I** and **N** and orange circles in panels **E**, **J** and **O**); patients/donors heterozygous for the *A3AΔ3B* deletion are represented by full black symbols in panels **A** though **J**. *p < 0.05; **p < 0.01; ***p < 0.001.

The two APOBEC3 enzymes which edit nuclear DNA, A3A and A3B, are ISGs. We therefore first compared *A3A* and *A3B* mRNA between SLE patients and healthy controls. *A3A* and *A3B* mRNA expression were ~ 10 to 15-fold (*p* < 0.01) and > 1000-fold (*p* < 0.001) higher respectively in SLE patients than in healthy controls (Fig. 1A,B). Higher A3B levels compared to A3A have been reported previously in cancer patients; nevertheless, the deamination activity of A3A is 100-fold higher than that of A3B^8,21–23^, explaining why such high levels of A3B, but not A3A, are compatible with cell viability. Analysis of RNA-Seq data from an independent cohort of 99 active SLE patients and 18 healthy controls (GEEO: GSE72509)^64^ confirmed a > 2.5-fold increase in *A3A* expression (*p* < 0.0001) and a threefold increase in *A3B* expression (*p* = 0.002) (data not shown). The fold-increase in RNA-Seq ranged from 0.5 to 6 for *A3A* and from 1 to 100 for *A3B*^64^. The less dramatic upregulation recorded with RNA-Seq likely reflects lower sensitivity of Illumina RNA-Seq chips and/or primer design, particularly considering the high sequence homology between *A3* genes.

*A3A* and *A3B* expression was higher in patients with elevated serum IFN-α and was weakly correlated with IFN-α levels (for *A3A*: r = 0.3285, *p* = 0.0163 and for *A3B*: r = 0.3227, *p* = 0.0196), as expected (Fig. 1F,G). It is possible that the heterogeneity and fluctuation of symptoms associated with SLE partially contribute to the weak correlation between *A3A* and *A3B* levels and IFN-α. Although only *A3A* and *A3B* edit DNA, we also measured the expression of two other ISGs, *A3G* and *RIG*-I, as well as *A3C*. All were upregulated in SLE patients compared to healthy controls (*A3G*: *p* = 0.0354; *A3C*: *p* = 0.0270; and RIG-I: *p* = 0.0012) and particularly in patients with detectable serum IFN-I levels (Fig. 1C–E). When SLE patients with detectable IFN-α in plasma were compared to patients with no detectable IFN-α, A3A (*p* < 0.05), *A3C* (*p* < 0.01), *A3G* (*p* < 0.01) and *RIG*-I (*p* < 0.01) expression were also more elevated (Fig. 1H–J). The observation that all were upregulated in SLE patients could be due to the fact that SLE is a multifactorial disease featuring many immunological dysregulations. Although *A3C* is generally not considered an ISG because it is much less responsive to IFN-I than *A3G* and *A3F*, its expression can be induced by IFN-I in PBMCs and in hepatocytes^15,16^. Furthermore, it is possible that other factors account for its upregulation in SLE patients. Nevertheless, since neither A3G nor A3C edit genomic DNA, it is unlikely that they contribute to lymphopenia. In line with this view, only *A3A* and *A3B* mRNA levels, were inversely correlated with lymphocyte counts (Fig. 1K,L) while *A3C*, *A3G*, and *RIG*-I mRNA levels were not (Fig. 1M–O), suggesting that genomic DNA editing by A3A and A3B may play a role in lymphopenia.

Taken together, these findings suggest that in SLE patients, the IFN-α response triggered by sustained cytosolic DNA due to oxidative mutations^39^ leads to persistant *A3A* and *A3B* expression among other ISGs. These deaminases may participate in DNA catabolism, but most of all, they could edit nuclear DNA. In this case, uridine resulting from cytidine deamination is removed by Uracyl DNA glycosylases (UNG), creating an apyrimidinic site. This mobilization can activate the DNA repair machinery, leading to C > T or C > G mutations which may be fixed^5–7^. Alternatively, juxtaposed apyrimidinic sites will generate DSBs causing cell death^5,6^. In both cases, A3-mediated deamination events may generate neoantigens, increasing the B-cell response against self-epitopes in a feed-forward loop and contributing to lymphopenia. Accordingly, *A3A* mRNA levels were higher in SLE patients with auto-antibodies against nuclear proteins (*p* < 0.05) and against dsDNA (Supplementary Fig. 1I) than in patients with no auto-antibodies, although statistical support was reached only for nuclear proteins. For *A3B*, mRNA levels were higher in SLE patients than in controls regardless of the presence of auto-antibodies (Supplementary Fig. 1J). For *A3C* and *A3G*, the levels of expression did not differ between SLE patients with or without auto-antibodies against nuclear antigens or against DNA (Supplementary Fig. 1 1 K and 1L). Although all *A3* enzyme mRNA measured in SLE patients were upregulated, these observations are strongly suggestive of a role for A3A- and A3B-induced DSBs in cell death and the induction of auto-antibodies in the pathogenesis of SLE.

Hierarchical clustering of patients based on disease severity showed significantly exacerbated *A3A*, *A3B* and *A3C* expression in patients with a postive SLEDAI (Supplementary Fig. 1A to 1D), as well as with the presence or severity of flares (Supplementary Fig. 1E to 1H). Notably, hydroxychloroquine, corticosteroids and immunosuppressive treatment failed to reverse elevated *A3A* or *A3B* levels back to background levels (Supplementary Fig. 1M to 1N), while the difference in *A3C* and *A3G* mRNA levels did not differ significantly between treated SLE patients, untreated SLE patients and controls (Supplementary Fig. 1O and 1P).

In the long run, sustained exposure to A3A and A3B mutational fuel could also generate oncogenic driver events^23,30–32^, providing a direct molecular rationale for the higher prevalence of certain tumors among SLE patients. We therefore searched for the presence of subclonal oncogenic driver mutations conforming to the preferred target for A3A and A3B, i.e. TpCpW^7,21,66^, in 2 oncogenes, *akt1* and the *TERT* promoter. We obtained sufficient sequences for all controls and 47 SLE patients. The threshold for distinguishing true subclonal mutations from error rate was set at 1 base call per 100 sequenced nucleotides. With these settings, we did not detect APOBEC3-mutations in *akt1* (not shown) nor in the *TERT* promoter (Supplementary Table 1). Because SLE patients in this cohort do not have cancer, we expected to find only a handful of APOBEC3-mutations, if any. Indeed, APOBEC3-mutations are generally not detected in non-cancerous, *TP53*- and *UNG*-positive cells^67^. It is however not excluded that subclonal mutations may be present at frequencies below the set threshold or elsewhere in the genome.

### High prevalence of the *A3AΔ3B* germline deletion among SLE patients

**Figure 2:**
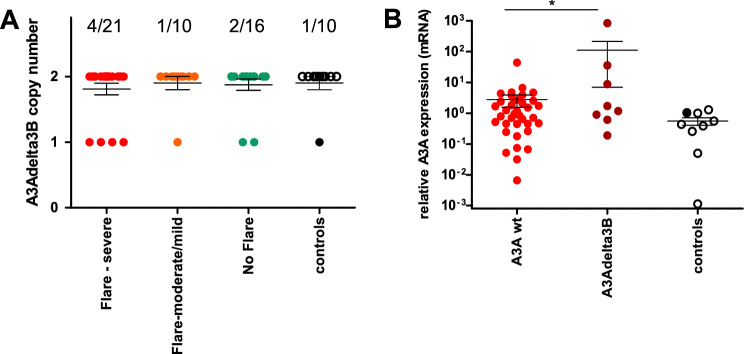
Prevalence and impact of the 29-kb deletion spanning *A3B* (*A3AΔ3B*) on *A3A* expression. (**A**) Prevalence of the *A3AΔ3B* deletion in SLE patients with severe or mild/moderate flares. (**B**) *A3A* relative expression in SLE patients with wild type *A3B* or with the *A3AΔ3B* deletion. *p < 0.05; **p < 0.01; ***p < 0.001.

A 29-kb germline deletion spanning the *A3B* locus generates a chimeric *A3A*-*UTRA3B* transcript (*A3AΔ3B*). *A3AΔ3B* is associated with increased *A3A* mRNA stability and protein expression^28^ and with a higher incidence of APOBEC3-mutations and cancer^6,23,29–32^. Copy number evaluation of *A3B* was performed by ddPCR to assess the prevalence of the *A3AΔ3B* polymorphism among SLE patients. The *A3AΔ3B* polymorphism was detected at the heterozygous state in 7/47 (14.9%) SLE patients and 1/10 (10%) controls. Although patients with severe flares (4/21 (19%)) had a slightly higher incidence of the polymorphism (Fig. 2A), statistical significance was not reached. As expected, the *A3AΔ3B* polymorphism was associated with higher A3A expression (*p* < 0.05) (Fig. 2B). *A3B*, *A3G*, *A3C* and *RIG*-I expression levels were unaffected by the single *A3B* allele (not shown).

### High IFN-I-induced A3A expression leads to SLE-cell death

**Figure 3:**
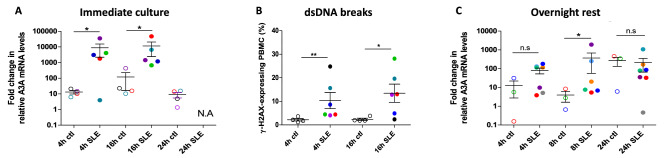
SLE patient cells do not survive in the presence of IFN-α. (**A**) PBMCs from SLE or healthy controls were isolated from blood and treated with IFN-α immediately after isolation and relative *A3A* expression quantified by qRT-PCR and normalized to the geometric mean of housekeeping genes *RPL13A* and *GAPDH* according to the Pfaffl method^65^ for each sample. The fold change relative to the untreated sample is reported. (**B**) Untreated cells from the same donors as in A were stained for DSBs (γ-H2AX) after 4 h and 16 h in culture. (**C**) PBMCs from SLE or healthy controls were isolated from blood, left to rest overnight and only then treated with IFN-α. Relative *A3A* expression was quantified by qRT-PCR and normalized to the geometric mean of housekeeping genes *RPL13A* and *GAPDH* according to the Pfaffl method^65^ for each sample. The fold-change in IFN-α-treated compared to non treated control 1 is reported. *p < 0.05; **p < 0.01; ***p < 0.001.

To further assess the role of detectable IFN-α and A3A in lymphopenia, freshly isolated PBMCs from SLE patients and healthy controls were put in culture with IFN-α for 24 h, mimicking flares. This setting recapitulates the fact that in SLE patients, but also in other chronic inflammatory conditions such as multiple sclerosis, flares often arise following an infectious episode, which likely triggers IFN-α. Initially, PBMCs from 5 SLE patients and 4 healthy controls were isolated and immediately exposed to IFN-α. By 4 h, IFN-α triggered a ~ tenfold increase in *A3A* mRNA, which was sustained throughout the time of the experiment in healthy controls (Fig. 3A). In SLE patients in contrast, IFN-α treatment increased *A3A* expression by more than 3 orders of magnitude (*p* < 0.05) (Fig. 3A). Such high *A3A* levels persisted 16 h post-treatment for all SLE patients, and by 24 h we could not measure *A3A* nor housekeaping genes, suggesting that SLE cells had died or were undergoing apoptosis (Fig. 3A). *A3A* and housekeeping gene mRNA could be quantified normally in cells from healthy controls treated with IFN-α, indicating that IFN-α per se did not induce massive cell death at these concentrations. In line with the high *A3A* mRNA levels in SLE patients, we recorded markedly higher levels of DSBs (γ-H2AX staining) in PBMCs from these SLE patients compared to healthy controls after 4 h (*p* = 0.0095) and 16 h (*p* = 0.019) in culture (Fig. 3B), indicating that higher *A3A* mRNA levels translate functionnally into increased DSBs and cell death.

Next, PBMCs from 7 SLE patients and 3 healthy controls were isolated, left to rest overnight before IFN-α treatment. In controls, *A3A* upregulation started around 8 h and reached only ~ 100-fold after 24 h (Fig. 3C). In SLE patients, IFN-α increased *A3A* mRNA by up to ~ 200-fold as early as 4 h post-exposure and the increase persisted at 8 and 24 h. Thus, *A3A* induction was much faster and stronger in SLE patients compared to healthy control cells even when cells were left to rest overnight after isolation (*p* < 0.05 at 8 h) (Fig. 3C).

These results nicely recapitulate the profound lymphopenia observed during flares in SLE patients, and point to A3A as a chief actor in the massive cell death that characterizes flares. They suggest that not only are basal *A3A* expression and DSBs persistently higher in SLE patients than in healthy individuals, but also that their cells are “primed” such that *A3A* expression is readily boosted to extremely high levels while it takes healthy cells much longer to trigger *A3A* to 100-fold lower levels. No cell could survive such high levels of A3A. This immediate and massive upregulation of *A3A* suggests the *A3A* promoter is poised in SLE cells.

### High prevalence of the TERT rs2853669 A > G polymorphism in SLE patients

**Figure 4:**
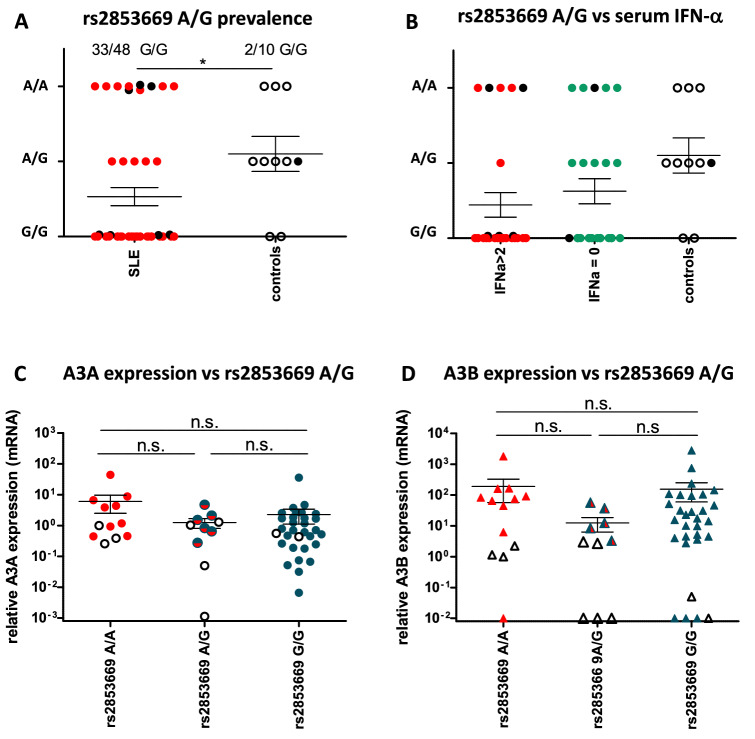
Prevalence of the TERT rs2853669 polymorphism in SLE patients. (**A**) Prevalence of the *TERT* promoter rs2853669 A/G alleles in SLE patients and controls. The number of GGAA sites at position − 245 from the TSS was assessed from the *TERT* promoter reads. (**B**) Prevalence of *TERT* promoter rs2853669 A/G alleles in SLE patients stratified according to serum IFN-α levels. Full black circles represent patients heterozygous for the *A3AΔ3B* deletion. (**C**) and (**D**) *A3A* (**C**) and *A3B* (**D**) expression levels in SLE patients based on rs2853669 G/A polymorphism. Full symbols are SLE patients and black open symbols are controls. *p < 0.05; **p < 0.01; ***p < 0.001.

Although we did not detect de novo somatic mutations attributable to A3 cytidine deaminase activity in the *TERT* promoter of our cohort, we found a surprisingly high prevalence of the germline polymorphism rs2853669 A > G among SLE patients compared to healthy controls (*p* = 0.0206). Of 48 SLE patients with *TERT* promoter sequences, 33 (68.75%) carried the rs2853669 A > G polymorphism in the *TERT* promoter on both alleles. Fifteen SLE patients (31.25%) had at least one GGAA Ets/TCF binding site (5 patients were heterozygous and 10 had wild-type rs2853669 A). In contrast, only 2 of 10 healthy controls (20%) were homozygous for rs2853669 A > G while all others harbored at least one copy of the rs2853669 A allele (5 were heterozygous and 3 were homozygous) (Fig. 4A).

The rs2853669 A > G is a common germline polymorphism which disrupts a prexisting Ets/TCF binding site located 245 bp upstream of the *TERT* TSS. Accordingly, the rs2853669 A > G polymorphism decreases *TERT* transcription in vitro. The rs2853669 A > G polymorphism has been investigated in cancers with *TERT* promoter mutations, where it may reverse their effect and cell immortalization^54–59^, but has not been investigated in inflammatory conditions to our knowledge. This germline polymorphism is not an APOBEC-mutation and accordingly, we found no relationship between the prevalence of the polymorphism and detectable IFN-α in serum (Fig. 4B) nor with *A3A* or *A3B* mRNA levels (Fig. 4C,D) or disease severity (severe or mild/moderate flares, SLEDAI > 10) (Supplementary Fig. 2A and 2B). Nevertheless, peripheral blood mononuclear subsets from SLE patients, and particularly terminally differentiated memory T cells, display shortened telomeres as a result of sustained immune activation. It was suggested that TERT activity is insufficient to compensate for telomere erosion and accelerated replicative senescence of immune cells in these patients^46–49,68^. Because the rs2853669 polymorphism decreases TERT activity, individuals carrying this polymorphim may have lower TERT activity and accelerated lymphocyte senescence. Although we could not test this hypothesis in our cohort, it is possible that repeated PBMC activation episodes during flares combined to a decreased inability to rescue activated immune cells from senescence could contribute to the frailty of SLE patients’ PBMCs and to lymphopenia.

## Conclusions

Our results document that multiple A3 enzymes, including IFN-induced *A3A*, *A3B* and *A3G*, and *A3C*, are strongly and persistently upregulated in SLE patients despite immunosuppressive treatment, and suggest a link between elevated baseline *A3A* levels, the *A3AΔ3B* polymorphism and disease severity. Since A3A and A3B can access the nucleus, these enzymes have every chance of editing nuclear DNA leading to DSBs. In this context, exposure to IFN-α leads to an immediate and massive upregulation of *A3A* expression, far beyond levels compatible with DNA repair, and ultimately to cell death. It is easily conceivable how high steady state A3A together with shortened telomeres due in part to the presence of the rs2853669 A > G polymorphism, might prime cells such that any further inflammatory signal leads to impelling *A3A* expression and massive cell death. Massive cell death in turn leads to exposure of nuclear antigens, fueling the inflammatory response and further incrementing the generation of auto-antibodies in a vicious cycle. These findings further underscore the need to include therapies targeting interferons and/or specifically A3 enzymes in the management of patients with lupus and probably other inteferonopathies^42^.

## Methods

### Patients and patient samples

Blood samples were obtained from 57 patients diagnosed with SLE according to the 1997 American College of Rheumatology criteria for SLE classification^69^. SLE patients were routinely followed at the French National Referral Center for Systemic Lupus Erythematosus, Groupement Hospitalier Pitié–Salpêtrière, Paris, France. SLE clinical characteristics, the Safety of Estrogens in Lupus Erythematosus National Assessment–Systemic Lupus Erythematosus Disease Activity Index (SELENA–SLEDAI)^62,63,70^, and the therapeutic regimen were recorded on the day blood was drawn. The class of lupus nephritis was recorded according to ISN/RPS-2003^71^. Routine testing to determine anti-dsDNA Ab titers Farr test (Trinity Biotech; cut-off value: 9.0 IU/mL), anti-ribonucleoprotein Abs (anti-RNP, anti-Sm, anti-SSA/Ro60, anti-Ro52/TRIM21, anti-SSB [Luminex FIDI, Theradiag]), and laboratory analyses (complement C3 levels (Optilite, Binding Site), complete blood counts, serum creatinine, proteinuria and hematuria) were run. The presence of a severe or mild/moderate lupus flare was recorded according to the SELENA-SLEDAI flare instrument^62^. For some analyses, patients were stratified according to the presence and severity of flares (i.e. no flare versus mild/moderate flare versus severe flare) or the SELENA-SLEDAI score as follows: SLEDAI = 0: non-active Lupus; 0 < SLEDAI ≤ 5: mild condition; 5 < SLEDAI ≤ 10: medium activity; SLEDAI > 10: severe activity. Serum-IFN-α biological activity, expressed in IU/mL, was determined by assessing the protection afforded by each patient’s serum to cultured MDBK cells challenged with vesicular stomatitis virus (VSV), as previously described^72^. Bioassay sensitivity (i.e. the lower limit of detection) was 2 IU/mL. Serum-IFNα activity in healthy individuals is undetectable, i.e. < 2 IU/mL^72^.

The study was approved by CPP Ile-de-France VI Ethics Committee. Samples were collected between November 2015 and October 2016. Ten sex-matched healthy controls whose blood was collected between June 15 and July 26, 2016 by the ICAReB platform at Institut Pasteur (Paris, France) were used for all experiments except for cell culture. All the participants gave written informed consent in the frame of the healthy volunteers CoSImmGEn cohort (Clinical trials NCT 03,925,272), which was approved by the CPP Ile-de-France I Ethics Committee (Jan 18, 2011). For cell culture experiments, blood from healthy volunteer blood donors from the Croix-Rouge Luxembourg was used. Written informed consent was provided by all patients and healthy donors. The research was carried out in compliance with the Helsinki Declaration.

Blood (2 × 7 mL) was collected on EDTA or PaxGene. Peripheral blood mononuclear cells (PBMCs) were isolated immediately by centrifugation, washed, split in two and dry pellets were stored at − 20 °C for DNA extraction and at − 80 °C for RNA extraction until use.

### RNA isolation and RT-qPCR

Total RNA was extracted from frozen PBMC pellets using Trizol. One microgram of total RNA was reverse transcribed with the QuantiTect reverse transcription kit (Qiagen). qPCR for *A3A*, *A3B*, *A3C* and *A3G*, *RPL13A* and *GAPDH* was performed in duplicate using Takyon Rox dTT Blue 2X Master Mix (Eurogentec), primers and Universal Probe Library probes as described in^16^ or the Applied Biosystems kit for *GAPDH*. For RIG-I, the following primers were used: F_RIG-I_: 5′-CTTTTTCTCAAGTTCCTGTTGGA-3′ and R_RIG-I_:5′-TCCCAACTTTCAATGGCTTC-3′, with UPL probe #79 (Roche). All genes of interest were normalized to the geometric mean of housekeeping genes *RPL13A* and *GAPDH* according to the Pfaffl method^65^ and the amplification efficiencies for these primers^16^. *A3A*, *A3B*, *A3C*, *A3G* and *RIG-I* relative expression levels were calculated by comparison with Healthy Donor C1.

### Next generation sequencing (NGS)

DNA was extracted from frozen PBMC pellets using the Epicentre kit and 1 μg of genomic DNA was amplified using Platinum HiFi Taq (Invitrogen). The *Telomerase Reverse Trancriptase* (*TERT*) core promoter was amplified using outer primers hTERT-out-F: 5′-AGTGGATTCGCGGGCACAGA^73^ and hTERT-out-R: 5′-GGCTTCCCACGTGCGCAGCAGGA^74^ and nested PCR primers : hTERT-in-F : 5′-GCACCCGTCCTGCCCCTTCACCT and hTERT-in-R : 5′-CAGCAGGACGCAGCGCTGCCTGA, spanning mutations C228T and C250T. *Akt1* intron 1 + exon 2, spanning mutation E17K, was amplified using primers Akt1-F: 5′-GCTGCCTGGCGAAGGTCTGACG and Akt1-R: 5′-CCTTGTAGCCAATGAAGGTGCC. PCR settings for both genes were: 5 min denaturation at 94 °C followed by 40 amplification cycles (94 °C 1 min, 63 °C 1 min and 68 °C 1 min) and a 10 min final extension at 68 °C. PCR products for the 2 genes were obtained for all controls and for 47 SLE patients for the *TERT* promoter and for 46 patients for *Akt1*. PCR products were gel purified, adaptors were added according to standard procedures for Illumina NextSeq 500 sequencing. For data processing, quality control was performed using FASTQC. All sequences were cut-off at a minimum quality PHRED score of 20. A minimum read length of 125 bp was selected. Reads were mapped against *TERT* (AF097365) and *Akt1* (NG_012188) reference sequences using the Geneious Software (V11.0.2) and Bowtie2 with standard settings for high sensitivity. SNPs were detected with the Geneious Software SNP detection tool with the following parameters: minimum coverage 500, minimum variant frequency 0.01, maximum variant p-value 1e-6 and minimum strand bias p-value 1e-5. Four SLE patients were excluded from further analyses because of insufficient reads mapping to the genes of interest.

### Detection of A3AΔ3B by ddPCR

*A3B* copy number was determined using the droplet digital PCR system (BioRad laboratories) in a duplex PCR targeting *A3B* and reference gene *RPP30* (RNase P, 2 copies per diploid genome^75^) for normalization. The *A3B* target region falls within the 29,5 kb deletion and within an 800 bp-fragment strictly specific to *A3B*. The following primers were used to generate a 60 bp fragment: F1_3B: 5′-GGCTGGACTCGCAGTCAC and R1_3B: AACAGCAGGGCTTAGGAACA together with *A3B*–specific probe UPL33 (Roche). Reaction mixtures (20 μL) comprised 10 μL 2× ddPCR Supermix (with dUTP; Bio–Rad), 900 nM of each *A3B*–specific PCR primer, 250 nM A3B–specific probe (UPL33) and 2 µL of 20× PrimePCR ddPCR reference assay (RNase P-HEX Bio-Rad). Genomic DNA (25 ng in 5 µL) was added to the ddPCR mixture and directly digested at room temperature with 3U of HaeIII (Thermo Scientific). Droplets were generated with a QX100 Droplet Generator (Bio–Rad) by mixing 20 μL of the assay reaction with 70 μL of droplet oil into the QX200 DG cartridge (Bio-Rad) according to the manufacturer’s recommendations, and were carefully transferred into a PCR plate. Thermal cycling conditions in a Bio-Rad iCycler (slow ramp rate, 2 °C per second) consisted in 10 min at 95 °C, 40 cycles of a two-step thermal profile of 15 s at 94 °C and 60 s at 59 °C, and a final step at 98 °C for 10 min. Droplets were counted using the QX200 Droplet Reader and *A3B* copy number assigned using QuantaSoft software (Bio-Rad)*.* Each 96 well plate included 8 randomly distributed `no template’ control reactions to check for the absence of contamination.

### Cell culture

Blood samples (30–35 mL) from an additional 12 patients with SLE (10 with inactive disease and 2 with active disease) and from 7 healthy donors from Croix Rouge Luxembourg, were collected on EDTA. No clinical data was available for these patients and healthy controls. PBMCs were isolated immediately by Lymphoprep density gradient centrifugation (Axis-Shield, Oslo, Norway), washed and seeded in 24-well plates (10^6^ cells/well) in RPMI 1640 medium supplemented with 10% FCS, 2 mM L-Glutamine, 50 µg/mL Penicillin and 50 µg/mL Streptomycin and 10 U/mL interleukin 2 (IL-2, Roche). Cells were treated with 750 U/mL IFN-α (PBL biomedical laboratories) either immediately for 4, 16 or 24 h (5 SLE patients and 4 healthy controls) or put to rest overnight and treated with IFN-α the following morning for 4, 8 or 24 h (7 SLE patients and 3 healthy controls). At the indicated time points, cells were harvested, washed and dry pellets were frozen at − 80 °C. RNA extraction and relative *A3A* and *A3B* expression levels (mRNA) were measured as for the other samples and normalized to the geometric mean of *RPL13A* and *GAPDH* according to the Pfaffl method^65^ for each sample (IFN-α-treated compared to the corresponding non treated control).

### Flow Cytometry

Cells from SLE patients or healthy controls cultured for 4 and 16 h without any stimulus were washed with cold PBS, fixed, permeabilized with methanol and stained with an AF647-conjugated mouse antibody against γ-H2AX (BD Pharmingen, clone N1-431) or AF647-conjugated Isotype control. Cells were analyzed using the MACSQuant cytometer and FlowJo version 10.6 (BD Bioscience).

### Statistical analyses

Statistical analyses were performed using GraphPad Prims v5 (GraphPad Software, San Diego, CA, USA). Mean/Median ± SEM were calculated. Groups were compared using a Mann–Whitney t-test for pairs and a Kruskal–Wallis with Dunn’s Multiple Comparison post test for more than two groups. Groups were considered to differ statistically if *p* < 0.05. Correlations between groups were tested using a Spearman test.

### Ethical clearance

The study was approved by the CPP Ile-de-France VI and CPP Ile-de-France I Ethics Committees for SLE patients and healthy volunteers CoSImmGEn cohort respectiviely. All donors provided written informed consent for this study. For cell culture experiments, blood from healthy volunteer blood donors from the Croix-Rouge Luxembourg was used. Written informed consent was provided by all donors. The research was carried out in compliance with the Helsinki Declaration.

## Supplementary Information

Supplementary Information
