# Supplementary material for: Sustained high expression of multiple APOBEC3 cytidine deaminases in systemic lupus erythematosus
Source: Sci Rep. 2021 Apr 12;11:7893. doi: 10.1038/s41598-021-87024-1 (PMC8041901; doi:10.1038/s41598-021-87024-1)
Supplement: Supplementary file 1 — Supplementary Information [file 41598_2021_87024_MOESM1_ESM.pdf]

Sustained High expression of multiple APOBEC3 cytidine deaminases in Systemic Lupus Erythematosus

Danielle Perez Bercoff[1], Hélène Laude[2,4], Morgane Lemaire[1], Oliver Hunewald[1], Valérie Thiers[3], Marco Vignuzzi[4], Hervé Blanc[4], Aurélie Poli[1], Zahir Amoura[5], Vincent Caval[6], Rodolphe Suspène[6], François Hafezi[1], Alexis Mathian[5], Jean-Pierre Vartanian[3,6†] and Simon Wain-Hobson[3†]

[1] Department of Infection and Immunity, Luxembourg Institute of Health, 29 rue Henri Koch, L-4354 Esch-sur-Alzette, Luxembourg. danielle.perezbercoff@lih.lu
[2] ICAReB platform, 28 rue du Docteur Roux 75724 PARIS CEDEX 15
[3] Molecular Retrovirology Unit, Institut Pasteur, CNRS, UMR 3569, 28 rue du Dr. Roux, F-75724, Paris cedex 15, France.
[4] Viral Populations and Pathogenesis Unit, CNRS UMR 3569, Institut Pasteur, 28 rue du Dr. Roux, F-75724, Paris cedex 15, France.
[5] Sorbonne Université, Assistance Publique–Hôpitaux de Paris, Groupement Hospitalier Pitié–Salpêtrière, French National Referral Center for Systemic Lupus Erythematosus, Antiphospholipid Antibody Syndrome and Other Autoimmune Disorders, Service de Médecine Interne 2, Institut E3M, Inserm UMRS, Centre d'Immunologie et des Maladies Infectieuses (CIMI-Paris), Paris, France
[6] Departement de Virologie, Institut Pasteur, 28 rue du Dr. Roux, F-75724, Paris cedex 15, France.
† These authors contributed equally.

Corresponding Author:
Dr Danielle Perez Bercoff
Department of Infection and Immunity, Luxembourg Institute of Health,
29 rue Henri Koch,
L-4354 Esch-sur-Alzette, Luxembourg.
Phone: +352 26 970 318
Email: danielle.perezbercoff@lih.lu

Supplementary Figures and Table

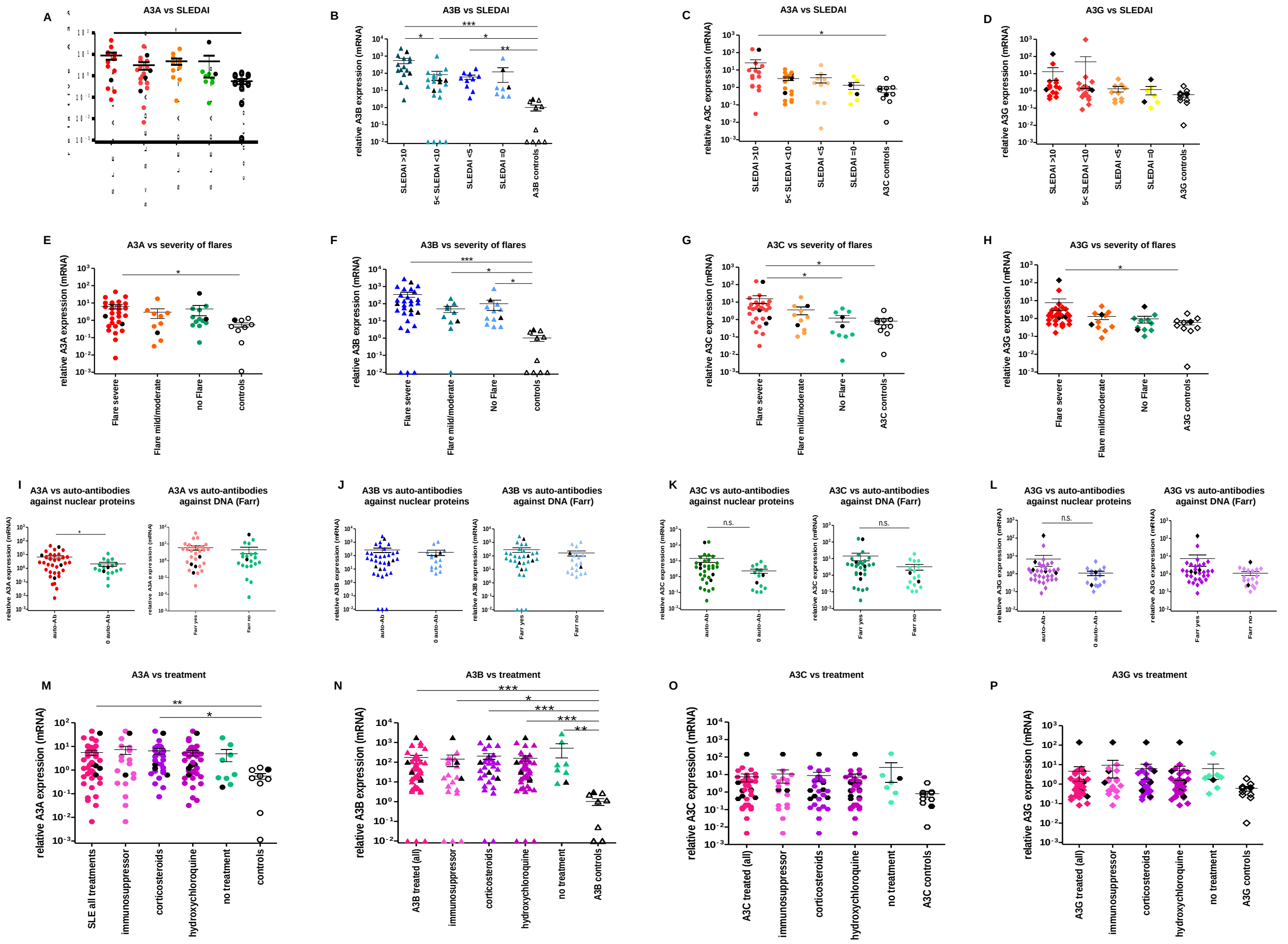

Supplementary Figure 1. *A3A*, *A3B*, *A3C* and *A3G* relative expression in SLE patients stratified by clinical status. A-D. *A3A* (**A**), *A3B* (**B**) *A3C* (**C**) and *A3G* (**D**) expression in relation to SLEDAI. **E-F.** *A3A* (**E**), *A3B* (**F**) *A3C* (**G**) and *A3G* (**H**) expression in relation to severity of flares. Patients were stratifed according to the presence and severity of flares or to the SELENA-SLEDAI Flare composite score as follows: SLEDAI = 0: non-active Lupus; 0<SLEDAI≤ 5: mild condition; 5< SLEDAI ≤10: medium activity; SLEDAI > 10: severe. **I-L.** *A3A* (**I**), *A3B* (J) *A3C* (**K**) and *A3G* (**L**) levels in patients with and without auto-antibodies against nuclear antigens and cellular DNA. **M-P.** *A3A* (**M**), *A3B* (N) *A3C* (**O**) and *A3G* (**P**)levels in relation to treatment. Relative A3A and *A3B* expression was normalized to the geometric mean of housekeeping genes *RPL13A* and *GAPDH* according to the Pfaffl method. Control C1 was used as calibrator for comparison and is therefore set to 1. Full black symbols represent patients heterozygous for the *A3AΔ3B* deletion. n.s.: not significant; n*: p<0.05; **: p<0.01; ***: p<0.001.

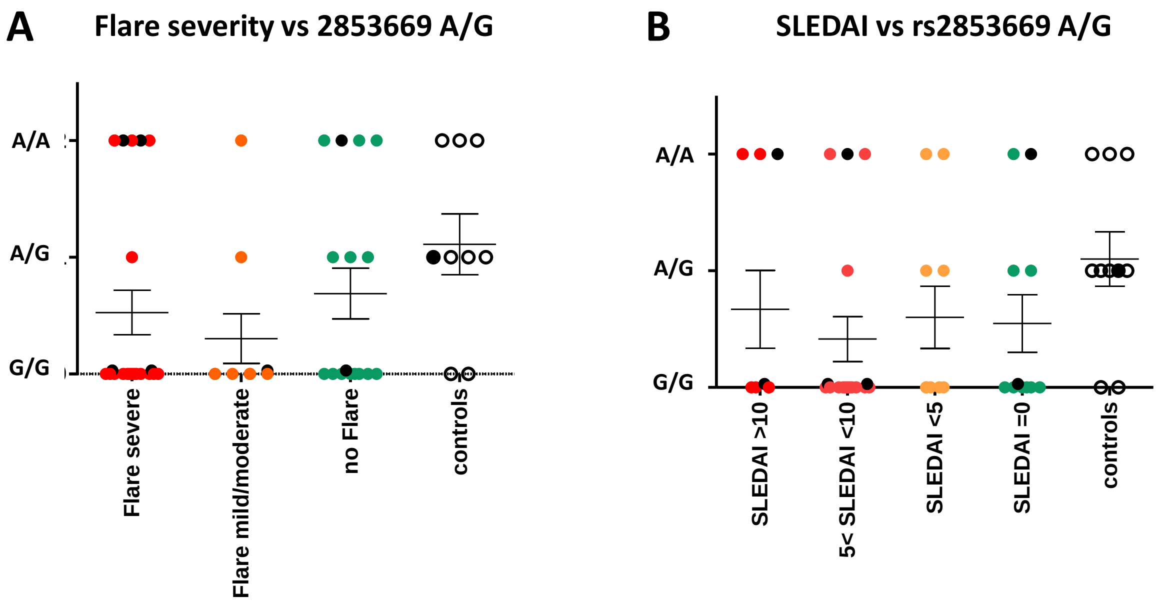

Supplementary Figure S2. **Prevalence of *TERT* promoter rs2853669 A/G alleles in SLE patients stratified according to flare severity (A) and SLEDAI (B).** Full black symbols represent patients heterozygous for the *A3AΔ3B* deletion. *: p<0.05; **: p<0.01; ***: p<0.001.

A TERT promoter sequence amplified

gtcgggg**cca ggccgggctc ccagtggatt** cgcgggcaca gacgcccagg accgcgc**tcc c**cacgtggcg
gagggactgg ggacccgggc acccgtcctg ccccttcacc ttccagctcc gcctcctccg cgcggacccc
gccccgtccc gacccc**tccc** gggtccccgg cccagcccc**c tcc**gggccct cccagcccct ccccttcctt
tccgcggccc cgccctctcc tcgcggcgcg agtttcaggc agcgctgc**gt cctgctgcgc acgtgggaag cc**

B

file_name	Position	Type	Reference	Allele	Count	Coverage	Frequency	Average quality	rs2853669 polymorphism	rs2853669
**controls**										
./HN3YNAFXX_C1_S61_cut (Reads) va	59.0	SNV	C	T	35565.0	35896.0	0.990778917	35 435 709 264 726 500	wt biallelic	rs2853669 A/A
./HN3YNAFXX_C2_S62_cut (Reads) va	59.0	SNV	C	T	3486.0	7508.0	0.464304742	35 421 399 885 255 300	polymorphism monoallelic	rs2853669 A/G
./HN3YNAFXX_C3_S63_cut (Reads) va	59.0	SNV	C	T	4886.0	7586.0	0.644081202	3 539 787 146 950 470	polymorphism monoallelic	rs2853669 A/G
./HN3YNAFXX_C4_S64_cut (Reads) va	59.0	SNV	C	T	48033.0	48228.0	0.995956706	35 454 729 040 451 300	wt biallelic	rs2853669 A/A
./HN3YNAFXX_C5_S65_cut (Reads) va	59.0	SNV	C	T	3111.0	3192.0	0.97462406	35 423 657 987 785 200	wt biallelic	rs2853669 A/A
./HN3YNAFXX_C7_S67_cut (Reads) va	59.0	SNV	C	T	25867.0	54657.0	0.473260516	354 282 290 176 673	polymorphism monoallelic	rs2853669 A/G
./HN3YNAFXX_C8_S68_cut (Reads) va	59.0	SNV	C	T	813.0	1501.0	0.541638907	3 533 825 338 253 380	polymorphism monoallelic	rs2853669 A/G
./HN3YNAFXX_C9_S69_cut (Reads) va	59.0	SNV	C	T	1832.0	2796.0	0.655221745	35 400 109 170 305 600	polymorphism monoallelic	rs2853669 A/G
**SLE patients**										
./HN3YNAFXX_S3_S3_cut (Reads) vari	59.0	SNV	C	T	1879.0	3912.0	0.480316973	3 529 856 306 546 030	polymorphism monoallelic	rs2853669 A/G
./HN3YNAFXX_S4_S4_cut (Reads) vari	59.0	SNV	C	T	9294.0	9343.0	0.994755432	35 401 226 597 805 000	wt biallelic	rs2853669 A/A
./HN3YNAFXX_S5_S5_cut (Reads) vari	59.0	SNV	C	T	8165.0	8200.0	0.995731707	35 385 303 123 086 300	wt biallelic	rs2853669 A/A
./HN3YNAFXX_S6_S6_cut (Reads) vari	59.0	SNV	C	T	33250.0	33423.0	0.994823924	353 532 030 075 188	wt biallelic	rs2853669 A/A
./HN3YNAFXX_S12_S12_cut (Reads) v	59.0	SNV	C	T	2484.0	2785.0	0.891921005	35 420 692 431 561 900	wt biallelic	rs2853669 A/A
./HN3YNAFXX_S13_S13_cut (Reads) v	59.0	SNV	C	T	28404.0	28546.0	0.995025573	3 536 899 732 432 050	wt biallelic	rs2853669 A/A
./HN3YNAFXX_S17_S17_cut (Reads) v	59.0	SNV	C	T	51889.0	52128.0	0.995415132	3 534 882 152 286 610	wt biallelic	rs2853669 A/A
./HN3YNAFXX_S20_S20_cut (Reads) v	59.0	SNV	C	T	23773.0	43794.0	0.542836918	3 537 349 093 509 440	polymorphism monoallelic	rs2853669 A/G
./HN3YNAFXX_S24_S24_cut (Reads) v	59.0	SNV	C	T	8856.0	18181.0	0.48710192	35 385 840 108 401 000	polymorphism monoallelic	rs2853669 A/G
./HN3YNAFXX_S25_S25_cut (Reads) v	59.0	SNV	C	T	31014.0	67159.0	0.46179961	3 541 694 073 644 160	polymorphism monoallelic	rs2853669 A/G
./HN3YNAFXX_S26_S26_cut (Reads) v	59.0	SNV	C	T	62577.0	62839.0	0.995830615	35 415 663 902 072 600	wt biallelic	rs2853669 A/A
./HN3YNAFXX_S28_S28_cut (Reads) v	59.0	SNV	C	T	21171.0	38966.0	0.543319817	35 439 232 912 946 900	polymorphism monoallelic	rs2853669 A/G
./HN3YNAFXX_S34_S34_cut (Reads) v	59.0	SNV	C	T	11090.0	23349.0	0.474966808	35 122 001 803 426 500	polymorphism monoallelic	rs2853669 A/G
./HN3YNAFXX_S35_S35_cut (Reads) v	59.0	SNV	C	T	7439.0	13477.0	0.551977443	3 526 737 464 712 990	polymorphism monoallelic	rs2853669 A/G
./HN3YNAFXX_S36_S36_cut (Reads) v	59.0	SNV	C	T	49246.0	50237.0	0.980273504	3 541 461 235 430 280	wt biallelic	rs2853669 A/A
./HN3YNAFXX_S38_S38_cut (Reads) v	59.0	SNV	C	T	17222.0	17296.0	0.995721554	3 536 081 755 893 620	wt biallelic	rs2853669 A/A
./HN3YNAFXX_S50_S50_cut (Reads) v	129.0	SNV	C	T	565.0	51116.0	0.011053291	3 436 106 194 690 260		
./HN3YNAFXX_S52_S52_cut (Reads) v	59.0	SNV	C	T	2072.0	2123.0	0.97597739	3 533 735 521 235 520	wt biallelic	rs2853669 A/A

Supplementary Table S1. Mutations and polymorphisms in a fragment of the *TERT* core promoter from SLE patients and healthy controls. A fragment of the *TERT* promoter was amplified by PCR and sequenced using Illumina 2000. **A.** The sequence is shown at the bottom of the list of mutations. Primers are underlined and bolded. The rs2853669 polymorphism as well as *TERT* promoter mutations -146C>T and -126C>T are highlighted in yellow. The putative Ets/TCF binding sites are underlined. Note that the reference sequence has the rs2853669 A>G polymorphism and has lost the Ets/TCF binding site. **B**. Position, mutation, Sequence Coverage, strand bias, variant frequency and the rs2853669 A/G status are reported.

SNV: single nucleotide variant; wt: wild type
